# Impact of a digital manual for guidance on malignant hyperthermia: patient education

**DOI:** 10.1186/s13023-022-02435-1

**Published:** 2022-07-15

**Authors:** Gislene Rodrigues, Pamela Vieira de Andrade, Joilson Moura dos Santos, José Luiz Gomes do Amaral, Helga Cristina Almeida da Silva

**Affiliations:** Brazilian Malignant Hyperthermia Unit - Discipline of Anaesthesiology, Pain and Intensive Care, Federal University of São Paulo, Rua Pedro de Toledo, 781, 04039-032 São Paulo, Brazil

**Keywords:** Patient education handout, Health education, Internet-based intervention, Telemedicine

## Abstract

**Background:**

Malignant hyperthermia (MH) is a rare, hereditary disease with a hypermetabolic response to volatile anesthetics/succinylcholine. Susceptible patients face difficulties due to a lack of knowledge about MH. As informational materials could increase knowledge and adherence to prevention/therapy, digital information about rare diseases validated for patients is needed. Our objective was to evaluate the following: (1) the impact of digital manuals on the knowledge/quality of life of MH patients and (2) access to MH services.

**Materials and methods:**

Fifty MH-susceptible patients filled out a virtual questionnaire twice (demographic/economic/clinical data, MH knowledge and impact on daily life, and SF-36 quality of life). Test groups 1 (n = 17) and 2 (n = 16) were evaluated 30 and 180 days after receiving a digital manual, and the control group (n = 17; without manual) was evaluated after 180 days. We collected the MH service data about the number of contacts.

**Results:**

Twenty-four (48%) patients reported problems in personal/professional life, sports, clinical/surgical/dental treatments, and military service, in addition to concerns about emergency care and complaints of sequelae. The percentage of correct answers in the second MH knowledge questionnaire increased for test group 2 (62% vs. 74.1%; unpaired *t* test, *p* < 0.01), was significantly greater in test groups 1 (68.1%) and 2 (74.1%) than in the control group (56.5%; Kruskal–Wallis, *p* < 0.05), and correlated with more time studying the manual and reports of MH-related problems (multiple regression, *p* < 0.05).

**Conclusions:**

The digital manual improved patients’ MH knowledge. Online contacts with the MH service increased, allowing greater information dissemination. As informational materials could increase knowledge/adherence to prevention/therapy, digital information about MH validated for patients should be implemented.

## Introduction

Malignant hyperthermia (MH) is a rare and inherited hypermetabolic response to volatile anesthetics/succinylcholine that occurs in 1:50,000 anesthesias in adults and 1:10,000 in children [1]. MH crisis is more common in men than in women (2:1) and in the second decade of life but affects all ethnicities, with a mortality rate of < 5–12% [1, 2]. Susceptibility to MH can be expressed as an idiopathic increase in creatine kinase (CK) and myopathies; exceptionally, crises are triggered by strenuous exercise/environmental heat [3]. MH-susceptible (MHS) patients can face several difficulties due to insufficient knowledge about their condition.

The dissemination of knowledge is essential for the expansion of self-management and greater capacity to deal with biopsychosocial aspects such as symptoms, treatment, and changes in lifestyle [4]. Informative health materials for patients, such as guidance manuals, can increase knowledge/adherence to prevention/therapy. The internet contributes to the dissemination of health information, but data on rare diseases are scarce. In 2017, there were 693 websites on rare diseases, but the information was difficult to evaluate by non-health care audiences [5]. Specific information about MH can be found on websites for health professionals (European Malignant Hyperthermia Group and Malignant Hyperthermia Association of the United States) and patient associations (ryr1.org). However, educational materials need to be adapted for patients and tested to verify their applicability/usefulness [6]. Thus, we aim to evaluate (1) the impact of a digital MH guidance manual on knowledge/quality of life and (2) access to MH service digital information tools.

## Results

Fifty patients, 24 (48%) of whom were male and 26 (52%) female with an average age of 41.46/13.89 years (range 18–71), were investigated due to a suspected susceptibility to MH in the patient (n = 10) or family members (n = 32), idiopathic CK elevation (n = 3) or myopathies possibly related to MH (n = 5). Sixteen of these patients reported one or more problems related to MH, affecting multiple aspects from their personal/professional life (n = 8 and 4, respectively) to health care (n = 4), sports (n = 4), dental care (n = 2), and military service (n = 2). Personal life problems referred to frequent pain/fatigue, fear of surgery/anesthesia/medications, death of a family member due to MH, the stress related to the need to warn their families about MH risk, and worries concerning medical insecurity regarding MH. Professional life was characterized by job instability resulting from frequent illnesses, pain/cramps, and exercise intolerance. Health care was compromised by frequent obstacles to scheduling surgeries, cancelled surgeries due to the unavailability of dantrolene, and insufficient medical knowledge about MH. Difficulty in participating in sports, especially high-performance activities, was associated with fear of unexpected problems. A few patients reported a fear of local anesthesia for dental care and the inability to perform some exercises during military service. Additionally, changes in family relationships were reported by eight patients, including shared concerns about anesthesia, efforts to deliver information about MH risk, and discrimination against MHS patients. Lifestyle modification occurred in 34 patients and was related to the increase in health care, moderation in sports, and concerns about accidents.

**Fig. 1:**
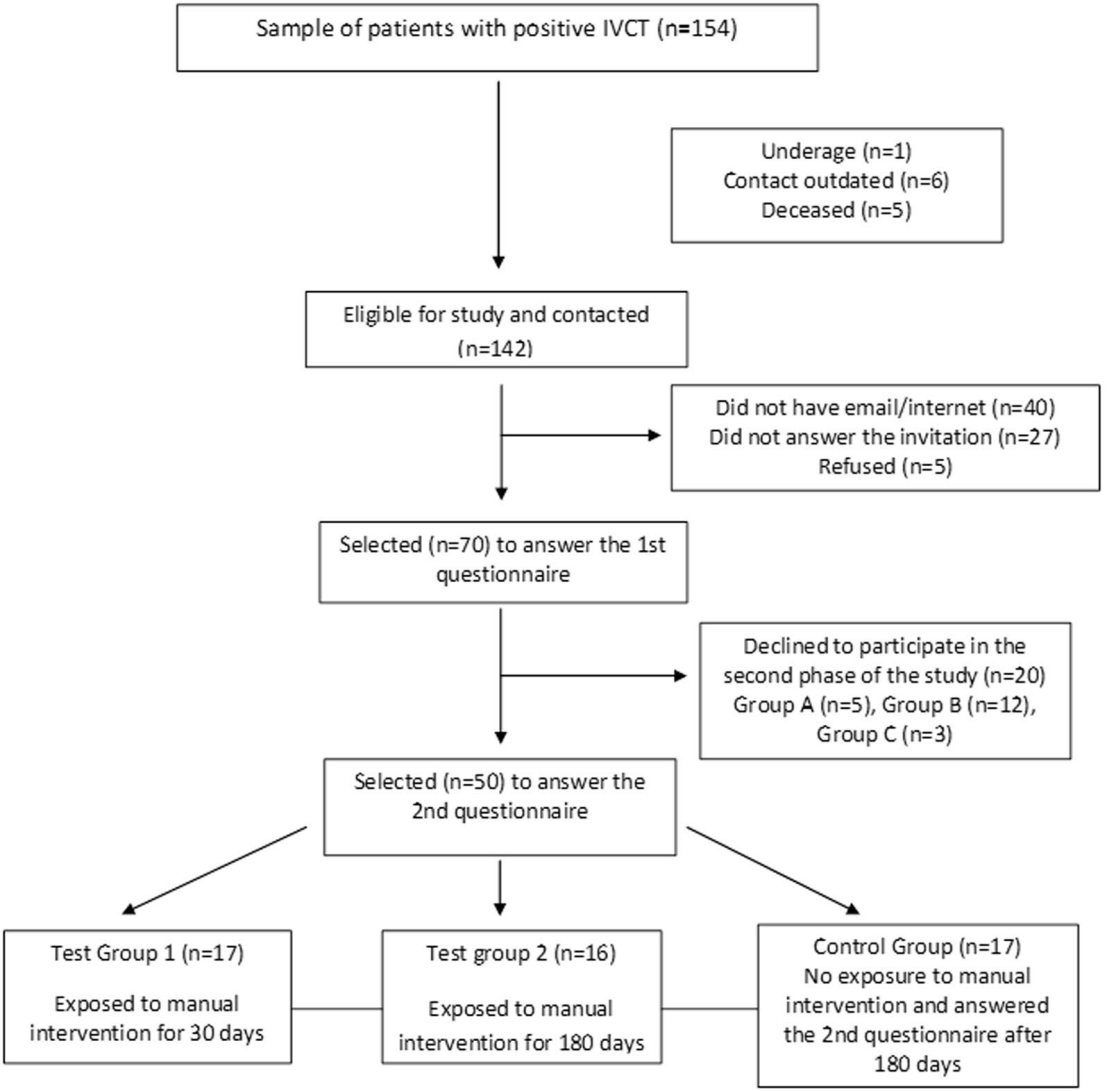
Flowchart of the participants

In the first assessment, the correct answer rate for the 10 questions about MH per patient was 60.17/18.63% (range 10–85%). The correctness rate per question ranged from 22% (question 10) to 94% (question 7), as follows: questions 1 (70%), 2 (86%), 3 (84%), 4 (74%), 5 (70%), 6 (82%), 8 (92%), and 9 (54%). Compared to TG2, TG1 presented a greater proportion of patients who did not undergo any type of follow-up related to MH, and a lower percentage did this follow-up in our center. There was no difference among the three groups for the other characteristics (Table 1; Fig. 1 A).

**Table 1:** Baseline characteristics of 50 patients

Variables	Test group 1 N = 17	Test group 2 N = 16	Control group N = 17	*p*
Age (mean/SD)	43.05/12.59	43.18/15.07	38.24/14.25	ns
Female (%)	47.06	50.00	58.82	ns
Higher educational level (%)	47.05	56.25	29.14	ns
No regular MH follow-up (%)	41.12	6.25	17.65	*p* < 0.019^a^ (95% CI[− 63.61, − 6.23])
Regular MH follow-up in our service (%)	47.06	81.25	64.71	*p* value < 0.043^a^ (95% CI [1.23, 67.15])
Economic classes A/B (%)	64.71	68.75	52.94	ns
MH death in the family (%)	29.41	43.75	23.53	ns
MH crisis in the family (%)	58.82	62.5	58.82	ns
MH related problems (%)	23.53	37.5	35.29	ns
Lifestyle changes (%)	29.4	12.5	35.29	ns
Changed family relationship(%)	11.76	18.75	17.65	ns
Increased number of medical appointment after MH (%)	29.4	6.66	18.75	ns
SF 36 domain 1 (mean/SD) (Functional Capacity)	74.41/28.77	85.66/18.21	83.48/26.50	ns
SF 36 domain 2 (mean/SD) (Physical Aspects)	80.88/34.83	85/31.05	76.66/40.61	ns
SF 36 domain 3 (mean/SD) (Pain)	60.42/32.39	69.94/26.59	80.25/25.32	ns
SF 36 domain 4 (mean/SD) (General Health Status)	70.03/22.82	70.16/30.41	80.6/19.49	ns
SF 36 domain 5 (mean/SD) (Vitality)	53.82/16.53	57.33/17.41	55.93/19.51	ns
SF 36 domain 6 (mean/SD) (Social Aspects)	66.17/31.80	80.83/16.28	75.83/26.92	ns
SF 36 domain 7 (mean/SD) (Emotional Aspects)	56.85/48.25	75.55/38.77	79.99/37.38	ns
SF 36 domain 8 (mean/SD) (Mental Health)	56.94/27.57	73.6/25.56	70.25/24.96	ns
Correct answers: 1st questionnaire (%)	62.72	62.02	55.93	ns
Correct answers: 2nd questionnaire (%)	68.11^b^	74.13^c^	56.58^d^	*p* < 0.006^e^

Legend: SD: standard deviation, MH: malignant hyperthermia, SF: Short Form Health Survey, ns: not significant. The chi-square test was used for percentages, and the unpaired t test for mean/SD values. ^a^Test Group 1 (TG1) versus Test Group 2 (TG2); ^b^*p*: not significant, questionnaire 1 versus 2; ^c^*p* < 0.002, 95% CI [− 17.69, − 5.12]. questionnaire 1 versus 2; ^d^*p*: not significant, questionnaire 1 versus 2; ^e^TG1 and TG2 groups versus control group (respectively *p* < 0.03, 95% CI [− 21.66, − 1.40] and *p* < 0.001, 95% CI [− 27.41, − 7.66]

In the second evaluation, the percentage of correct answers was higher in the TG1 and TG2 groups than in the control group (Table 1; Fig. 1B). TG1 and TG2 had no significant differences between the first and second assessments in the percentage of patients who reported problems related to MH and changes in lifestyle, in the number of medical consultations, or in the mean score of the SF-36 subdomains, with the exception of the vitality subdomain, which decreased in TG2 (53.82 vs. 42.82, *p* < 0.008, 95% CI [3.08, 16.92]). In both groups (TG1 and TG2), there was an increase in the percentage of patients who reported changes in family relationships (TG1 18.18 vs. 39.39, *p* < 0.02, 95% CI [− 38.39, − 4.03]; TG2 14.17 vs. 41.18 *p* < 0.005, 95% CI [− 44.30, − 8.64]). Most patients found the manual useful (TG1 76.47%, TG2 100%).

**Fig. 2:**
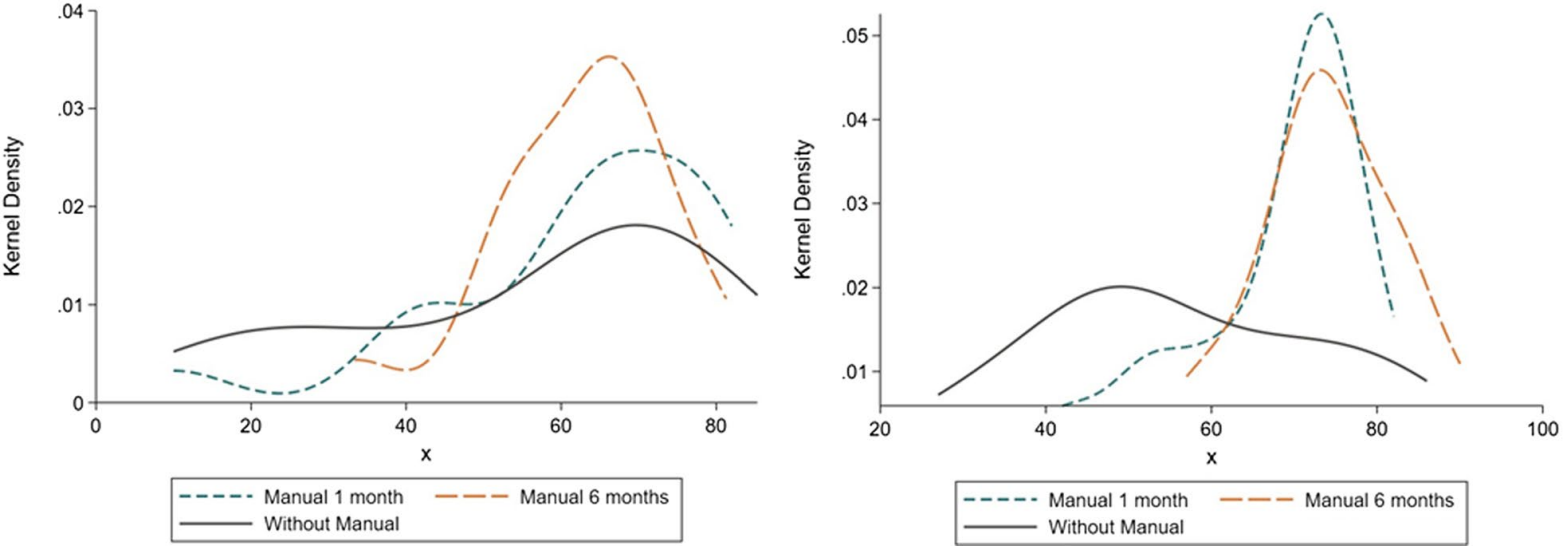
Kernel density of MH test scores for questionnaire 1 (**A**: left) and 2 (**B**: right)

The kernel densities of the scores for each group, in the first assessment, had a similar pattern (Fig. 2A). In the second evaluation, the curves for TG1 and TG2 were much more similar; a significant portion of the distributions overlapped, while the difference between these groups and the control group became evident (Fig. 2B). In the first assessment, multiple regression analysis detected female sex, economic classes A/B, changes in lifestyle after a diagnosis of MH, and greater vitality in the SF-36 subdomain as predictors of a higher percentage of correct answers on the MH knowledge test (Table 2). In the second assessment, predictors of a higher percentage of correct answers on the MH knowledge test were TG2, problems related to MH, and a higher percentage of correct answers on assessment 1 (Table 2).

**Table 2:** Explanatory variables and correct answers on the MH knowledge questionnaire (regression coefficients (standard error))

Explanatory variables	% correct answers (evaluation 1)	% correct answers (evaluation 2)	
Sex (female = 1)	**16.95 (6.60)a**	6.43 (5.64)	2.46 (3.79)
30 days with manual	7.68 (6.53)	2.94 (4.84)	5.09 (4.83)
180 days with manual	5.98 (5.54)	**11.07 (4.95)**^**a**^	**12.35(4.19)b**
Higher educational level	− 7.12 (5.74)	1.41 (4.46)	− 0.77 (3.84)
Regular follow-up for MH (evaluation 1)	− 8.79 (7.34)	5.01 (3.90)	–
A/B economic classes	**13.67a** **(6.59)**	3.44 (6.57)	3.68 (4.17)
MH death in the family	2.01 (6.28)	1.57 (5.91)	–
Indication for investigation (evaluation 1)	− 4.58 (8.09)	− 7.75 (5.50)	–
MH-related problem (evaluation 1)	2.58 (5.98)	− 2.43 (4.60)	–
Impact on personal life (evaluation 1)	4.17 (6.46)	3.63 (5.76)	–
Lifestyle changes (evaluation 1)	**18.78 (5.53)**^**b**^	− 0.28 (4.26)	–
Medical appointments (evaluation 1)	− 4.71 (7.41)	− 1.89 (5.67)	–
Changes in family relationship (evaluation 1)	− 3.65 (7.02)	1.88 (5.91)	–
SF 36 - vitality (evaluation 1)	**0.27 (0.12)**^**a**^	− 0.07(0.15)	–
MH related problems (evaluation 2)	−	**10.95 (3.94)**^**b**^	**9.49 (2.64)**^**b**^
Lifestyle changes (evaluation 2)	–	12.05 (6.29)	10.21 (5.16)
Medical appointments (evaluation 2)	–	1.29 (6.01)	1.52 (5.10)
Changes in family relationship (evaluation 2)	–	− 6.56 (4.32)	− 4.94(3.28)
SF 36 - vitality (evaluation 2)		0.21 (0.14)	0.11 (0.11)
Percentage of correct answers (evaluation 1)	–	**0.34 (0.12)b**	**0.35 (0.10)b**
Constant	23.79 (8.79)^b^	29.65 (11.15)^b^	29.77 (8.49)^b^
R-square	0.53	0.71	0.63

Legend: MH: malignant hyperthermia, SF: Short Form Health Survey. ^a^*p* < 0.05. ^b^*p* < 0.01

**Fig. 3:**
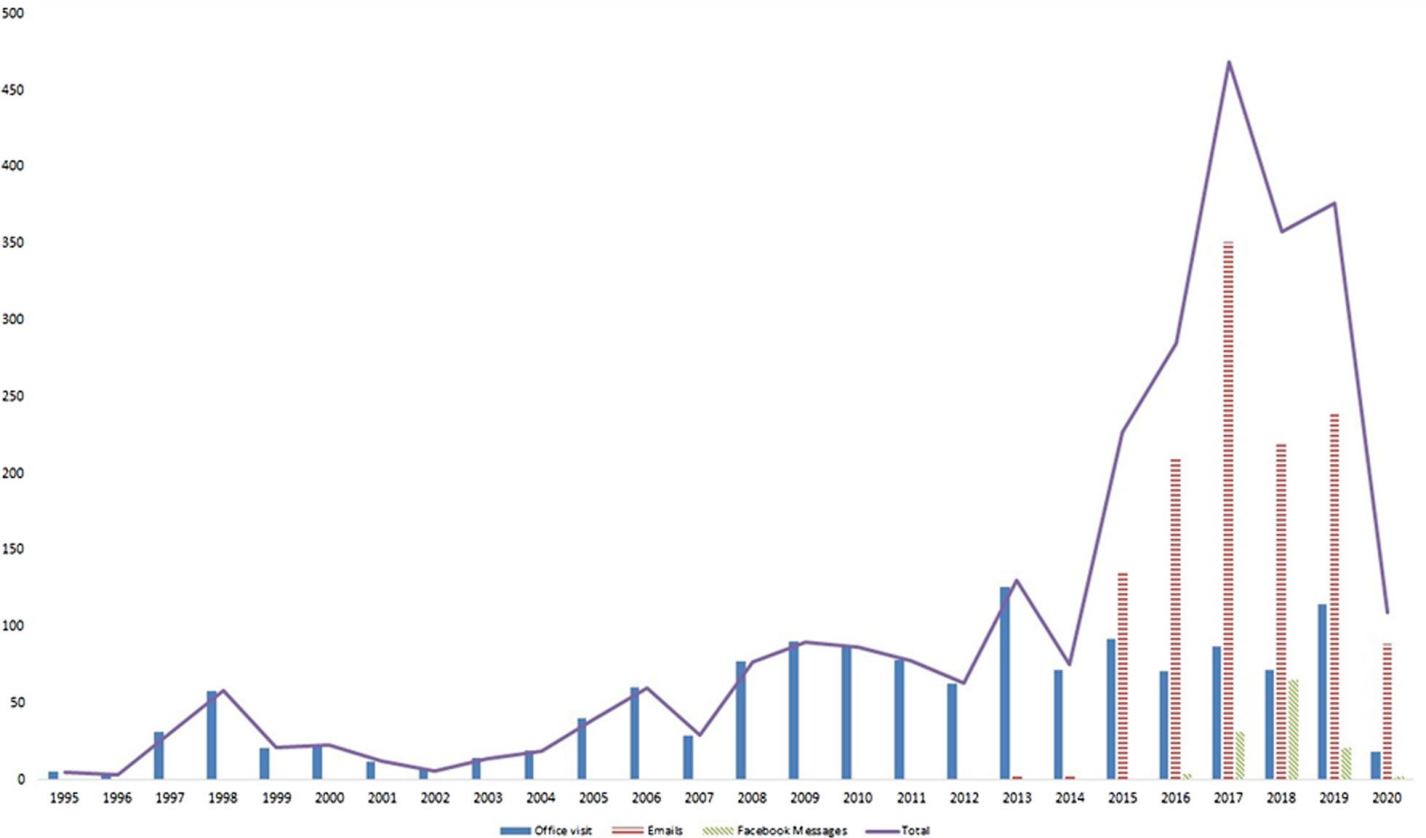
Medical consultations, Emails, and messages via Facebook per year. Implementation dates were 2010 for email and 2016 for Facebook/website

From January 2010 to June 2020, we received 1,254 emails from patients and 50 emails from health care professionals. Most emails dealt with consultations/exams (n = 878). There was increased use of this communication tool during the study, with a peak immediately after the creation of the website/Facebook page (Fig. 3). The 1254 emails were sent by 192 individuals: 50 participants of this survey (327 emails: 6.54 emails per participant) and 142 nonparticipants (927 emails: 6.52 emails per participant).

The website cedhima@unifesp.br received 9,934 visits (2016–June 2020), and the FAQ section was the most visited (n = 2,202). On Facebook (2016–June 2020), 164 friendship requests were sent and accepted from patients or health care professionals in Brazil and other countries in Latin America, North America, Africa, and Asia. Posts on the Facebook page had 180 reactions, 36 shares, 41 comments, and 2,806 visits.

Through Facebook or Messenger, 162 messages were received from 43 individuals. Twelve participants in this study sent 49 messages (3.58 per participant), and 31 individuals who did not participate in this study sent 113 messages (3.64 per participant). These messages concerned a wide variety of subjects, the most relevant being the scheduling of appointments (n = 41) and updating of registration data (n = 17).

## Discussion

In this study, we demonstrated that a digital manual for MH guidance increased patients’ knowledge about MH without interfering with their quality of life. In addition, we detected a growing volume of access to our group’s digital MH information tools.

There was a predominance of individuals in the third decade of life, similar to previous studies on health education, where there was a decrease in access among elderly people who used digital information technology, despite the interest in seeking information about chronic diseases [7, 8]. A large part of the sample had higher education levels and belonged to the middle or upper classes; they were possibly overrepresented because access to the internet/email was part of the inclusion criteria. In the United States, middle-aged adults and senior individuals with higher education levels were more likely to use digital media when seeking health information, while there was a decrease in access among senior individuals aged 75 and over and among adults with low income, low education levels, and unemployment [7, 8]. However, in our study, the education level did not influence the degree of correctness of the MH knowledge questionnaire, while socioeconomic level positively influenced the first assessment only, suggesting that the manual contributed to the leveling field in terms of knowledge.

A large part of the sample had problems related to MH, changes in family relationships, and lifestyle modifications. Systematic studies of this population have shown not only sequelae of an MH crisis but also other alterations, such as the potential tendency to bleed [3]. Susceptibility to MH, in addition to the possibility of an anesthetic crisis, also causes other problems that are sometimes underdiagnosed, such as exercise intolerance, cramps, and muscle pain [9]. These problems could have contributed, in the SF-36 quality of life questionnaire, to the lower initial scores in the vitality subdomain. The mental health subdomain also had lower scores in TG1, although with no significant difference between groups. These changes could reflect low vitality, as well as patients’ concerns about the risk that MH poses for them and their families. We can speculate that knowledge about MH has caused this impact by generating greater concerns about the harm caused by this disease. There could be a concern about this learning tool causing too much anxiety or even depression. However, the SF-36 domain 8, related to mental health, includes questions related to anxiety/depression, and there was no difference in responses from individuals with or without the manual. It is necessary to investigate whether this situation changes over time, considering that six months could be a short time for this analysis.

There was good baseline knowledge about MH, except regarding the questions on which less systematic information is available, related to restrictions with medications that could compromise skeletal muscles and activities of daily living that could lead to muscle damage [10–12]. These issues are addressed in the manual, with an emphasis not on the prohibition but on the care with medications and risky activities, which involves notifying physicians and sports advisors.

The better performance of female participants in the first assessment may reflect their greater search for knowledge, especially in digital media, according to a previous study’s suggestion that women are more interested in information about their health and that of their family members; in addition, females predominate in health education studies regardless of age [13]. On the other hand, the better initial performance of economic class A or B participants may reflect better access to information sources [14]. Likewise, greater knowledge about MH in the group with greater vitality according to the SF-36 may result from a greater ability to seek additional sources of knowledge. A higher degree of correct answers in the group that underwent lifestyle changes after the diagnosis of MH was an interesting finding in the first assessment, which could reflect that the group sought to obtain more information about MH and therefore made lifestyle changes. However, these four factors did not affect the second assessment. The percentage of correct answers in the first assessment, used as a variable in the second regression, carried the effect of prior knowledge, helping to further isolate the effect of the manual on the result. For this reason, possible characteristics such as sex and economic class were no longer significant in the second evaluation. Density analysis revealed that the manual contributed to making the knowledge about MH more uniform, improving patients’ performance, and thus reducing the dispersion of the scores in the second questionnaire.

In the second evaluation, groups that received the manual had a higher number of correct answers than the group without the manual, but the present research did not detect an impact of the manual on the other evaluated characteristics, suggesting that the manual did not cause harm in the aspects analyzed. We emphasize the better performance regarding correct answers for the group that spent more time with the manual, which could be explained by the possibility of repeating the manual reading more times and retaining the information in a better way. In addition, in the second assessment, a higher percentage of correct answers on the MH knowledge test was predicted for problems related to MH. The present research does not allow us to define whether patients with more knowledge were more aware and attentive to the appearance of MH-related problems or if, alternatively, patients with MH-related problems studied the manual more deeply before the second assessment.

This study had some limitations linked to the sample size and the length of the longitudinal follow-up. Although the calculation of the sample size and the six-month interval was sufficient to show an improvement in knowledge, they may have been insufficient to show an impact on quality of life, leading to the possibility that the regression could be underpowered. The patients not included in the survey because they refused to participate in the second questionnaire should be analyzed in future studies to investigate the reasons for noncompliance and the development of specific educational measures for them.

The increase in the number of e-mails received by our center during this study reflects the adherence of patients to this communication tool, especially in the context of rare diseases such as MH, which are managed by specialized centers sometimes far from patients’ homes [15, 16]. The considerable number of visits to the website reinforces the positive influence of the internet on health education, where the website is a model of written communication that stands out in transmitting information to patients to improve self-care [17, 18].

Additionally, the use of Facebook had great repercussions on our study. There has been an increase in the use of Facebook to disseminate health education through the internet [19]. Social media has the potential to improve communication with patients and the dissemination of health education [20]. The majority of Facebook friends in our study were female, in accordance with previous studies on health information transmitted via the internet and social networks [21]. There are benefits of using social media to obtain health-related information for individuals in various segments of society, regardless of age, education level, and ethnicity [22].

The internet provided the development of a channel of communication with patients, allowing a growing number of people to search for information through the website, Facebook, and e-mail. At the same time, the digital manual of guidance on MH for patients proved to be a good tool for information about MH, resulting in increased knowledge about the disease without compromising quality of life, particularly in the group that spent more time with the manual and had problems related to MH. As informational materials could increase knowledge/adherence to prevention/therapy, digital information about this rare disease validated for patients should be implemented.

## Methods

We analyzed 50 MHS patients followed at our center, divided them into three groups, and interviewed them in two stages, with data collection from November 15, 2016, to July 17, 2019. In the timeline of this experimental design, we only grouped and presented in the article the people who have clearly participated both in the first and the second questionnaire survey. The study was controlled, randomized, and prospective, with an intervention (digital guidance manual). The inclusion criteria were as follows: minimum age of 18 years, access to e-mail/internet, and MH susceptibility confirmed by a positive in vitro contracture test (IVCT) according to the European Malignant Hyperthermia Group protocol [23]. The study was approved by the Research Ethics Committee of the institution (number 1.238.764) and was carried out in accordance with the code of ethics of the Helsinki Declaration, and informed consent was obtained from all participants.

Among 154 patients with a positive IVCT, 70 completed the electronic consent form and answered the first digital questionnaire. Fifty patients answered the two digital questionnaires and completed the survey (Fig. 1). These 50 individuals belonged to 36 families, with an average of 2.7 participants per family (range 1–6) and were divided into three groups: (A) test group 1 (TG1): 17 patients evaluated with a second questionnaire 30 days after receiving the digital manual; (B) test group 2 (TG2): 16 patients evaluated 180 days after receiving the digital manual; and (C) control group (CG): 17 patients who did not receive the digital manual and were reassessed 180 days after the first questionnaire. We chose the 180-day interval to conduct the questionnaire on TG2 and CG according to a previous study by our group that showed a decrease in 30-day learning retention after 180 days of theoretical classes [24]. The questionnaire applied at the first time consisted of (a) demographic/clinical data; (b) economic classification into eight categories according to decreasing consumption patterns/potentials (A1/A2/B1/B2/C1/C2/D/E) [25]; (c) questions about problems related to MH (personal/professional life, military service, sports, medical/dental care, others) and changes in family relationships and lifestyle; (d) 10 questions about MH (10 points for each correct question); and (e) the SF-36 quality of life questionnaire (zero represents the worst situation and 100 represents the best one) [26]. The text of the complete questionnaire is available as supplementary material in a previous article by our group [27]. The questionnaire applied the second time consisted of the same previous items: (c) questions about problems related to MH and changes in family relationships and lifestyle, (d) 10 questions about MH, and (e) the SF-36 quality of life questionnaire.

The questionnaire to assess MH knowledge was developed in our center by two physicians and a nurse with experience in MH and presented good acceptance with a 60% overall rate of correct answers in a previous survey [27]. There were two open-ended questions (questions 1 and 10) and eight yes/no or multiple choice questions, as follows: (1) What are the signs of an MH crisis? (right answers included abnormal heart/respiratory frequencies, muscle rigidity, hyperthermia, cyanosis, elevated exhaled carbon dioxide, sweating, and blood pressure instability); (2) What is the origin of MH disease? (genetic origin and inherited from parents); (3) When does an MH crisis occur? (inhalation of anesthetics such as halothane and use of the succinylcholine muscle relaxant); (4) How is MH treated? (interruption of surgery with triggering anesthetics and administration of dantrolene sodium); (5) Who is at risk for MH? (the person who suffered an MH crisis and their family members); (6) How is MH prevented? (preanesthetic evaluation, monitoring of expired carbon dioxide during anesthesia, availability of dantrolene in surgical centers, and training of health care professionals); (7) Can an MHS patient undergo surgery? (yes); (8) Can an MHS patient receive local anesthesia during dental treatment? (yes); (9) Do MHS patients have any medication restrictions? (yes); and (10) Does an MHS patient have any restrictions in their daily/professional life? (yes: restrictions regarding excessive heat/physical exertion and the use of illicit drugs). The manual was based on the following: (1) A literature review about the impact of MH on daily life and (2) the demands of MHS patients. This manual addressed prenatal diagnosis, dental treatment, physical exercise, management of preexisting diseases, exogenous intoxications, illicit drugs, and medications [28]. Before the beginning of data collection and digital manual delivery, we performed a pilot for in-person physical manual delivery and reviewed it with the patient to check for his or her understanding and to ask if there were additional questions. All patients included in the data collection had at least four years of formal education and received our email contact, which was always available and monitored to resolve any issues.

The three groups received the same information in the initial medical consultation and had free access to the email (cedhima@hotmail.com), website (cedhima.unifesp.br), and Facebook page of the MH service. The website followed the methodology previously described and the Health on the Net Foundation Code of Conduct for websites [29, 30]. Validation of the content and appearance of the website was carried out by health professionals from our center and by a pilot group of MHS patients. The website contained the following sections: user registration, quick search, information for laypeople and health professionals, legislation, basic MH concepts (definition, diagnosis, and treatment), MH in the family (care, recommendations, myths, and truths), news (articles, news, and interviews), contact, FAQ (frequently asked questions), useful links, videos, and testimonials.

There is a paucity of detailed data on the impact of MH on daily life, knowledge about the disease and quality of life in susceptible patients. To investigate the relationship between these variables, ideally, we would need previous studies on these variables to perform an accurate sample calculation. In the absence of these data, in the present study, the sample calculation considered the data from previous surveys by our group that showed an average increase of 24% in the percentage of correct answers after educational activities [24]. To replicate this finding of MH knowledge before/after an intervention, with an alpha significance level of 5% and a statistical power of 80%, at least four individuals per group would be needed during the study period. Randomization, according to a table with a sequence of random numbers generated in Excel, was performed by family and not by individuals to surpass the information exchange inside families. The random allocation sequence was generated by HCAS; GR enrolled participants and assigned participants to interventions.

Data were tabulated and analyzed for normal distribution with the Kolmogorov–Smirnov distance test. Categorical data were described as absolute/relative (n/%) frequencies. Noncategorical data were expressed as the mean/standard deviations, median/quartiles 25-75%, or percentages. Differences between groups were calculated using Mann–Whitney U and t tests, with *p* < 0.05 considered significant. Multiple regression (ordinary least squares) was performed to detect which characteristics of the patients, including exposure to the MH manual, could be predictors of the percentage of correct answers in the MH knowledge test.

## Data Availability

The datasets used and/or analyzed during the current study are available from the corresponding author on reasonable request.

## Abbreviations

CK: Creatine kinase
FAQ: frequently asked questions
IVCT: In vitro contracture test
MH: Malignant hyperthermia
MHS: Malignant hyperthermia susceptible
TG: Test group
CG: Control group
